# Binding affinity of five PBPs to *Ostrinia* sex pheromones

**DOI:** 10.1186/s12867-017-0079-y

**Published:** 2017-02-07

**Authors:** Tiantao Zhang, Yaqi Sun, Kevin W. Wanner, Brad S. Coates, Kanglai He, Zhenying Wang

**Affiliations:** 1 State Key Laboratory for the Biology of the Plant Diseases and Insect Pests, Institute of Plant Protection, Chinese Academy of Agricultural Sciences, No. 2 West Yuanmingyuan Road, Beijing, 100193 China; 2 College of Bioscience Technology, Shenyang Agriculture University, Shenyang, China; 3 Department of Plant Sciences and Plant Pathology, Montana State University, Bozemon, MT 59717 USA; 4 United States Department of Agriculture, Agricultural Research Service, Corn Insects and Crop Genetics Research Unit, Iowa State University, Ames, IA 50011 USA

**Keywords:** Pheromone binding protein, Binding, Sex pheromone, Docking, Mutant

## Abstract

**Background:**

Pheromone binding proteins (PBPs) of male Lepidoptera function in chemical communication, mate attraction and recognition. Directional selection was previously predicted between PBP3 orthologs of *Ostrinia furnacalis* and *Ostrinia nubilalis* were interpreted as being involved in sexual isolation.

**Results:**

In vitro assays show that recombinant male *Ofur*PBP3 bound *O. furnacalis* sex pheromones, *Z*-12-tetradecenyl acetate (*Z*12-14:OAc) and *E*-12-tetradecenyl acetate (*E*12-14:OAc), as well as to ECB pheromones *Z*11- and *E*11-14:OAc. Recombinant *Ofur*PBP4 and *Ofur*PBP5 bound *E*11- and *Z*11-14:OAc with greater affinity compared to *Z*12- and *E*12-14:OAc, and *Ofur*PBP4 incapable of binding with *E*12-14:OAc. In silico molecular docking predicted *Ofur*PBP3 residues Phe12, Ile52, Leu94, Ile113 within a hydrophobic ligand-binding pocket and may participate in *E*12- and *Z*12-14:OAc binding. Independent site-directed mutagenesis experiments demonstrated that Ser12, Asn52, Arg94, and Asn113 residues variants caused an approximately 1.7- to 4.6-fold reduction in *Ofur*PBP3 affinity for *Z*12- and *E*12-14:OAc, and a 2.7- to 8.4-fold decrease in affinity towards *E*11- and *Z*11-14:OAc.

**Conclusions:**

Five PBPs of *O. furnacalis* play important functions in *Ostrinia* pheromones binding. These four amino acids may play a role in binding of sex pheromone, but this study does not address questions regarding specific response between males of *O. furnacalis* and *O. nubilalis*. Additional studies are required determine the role, if any, PBPs play in the evolution of sex pheromone communication.

**Electronic supplementary material:**

The online version of this article (doi:10.1186/s12867-017-0079-y) contains supplementary material, which is available to authorized users.

## Background

The sex pheromone detection by male moths (insect Order Lepidoptera) is highly sensitive and capable of detecting volatile hydrocarbons at low concentrations, as well as discriminating among stereoisomers and pheromone blends [1]. Female moths produce and emit volatile sex pheromones from pheromone glands located in distal segments of the abdomen, and perception can cause conspecific males to be attracted from a distance of 100 m [2]. The specificity of male reception and response to female sex pheromones is mediated by olfactory response neurons (ORNs) located in antennal trichoid sensilla. Detection of female pheromones can induce physiological or behavioral changes in males following downstream signal transduction events mediated by the ORN [3]. The first step of pheromone detection is the transport of the hydrophobic pheromone across the hydrophobic lymph of the trichoid sensillum. Pheromone binding proteins (PBPs) are a subgroup of the odorant binding protein (OBP) family that have been reported in many species of Lepidoptera [4], and are hypothesized to be essential in environmental detection of sex pheromones by male moths [5]. PBPs are small, water-soluble, extracellular proteins that are found in the lymph surrounding the dendrites of pheromone-sensitive ORNs [6]. PBPs bind short to medium chain length fatty acid derivatives that enter the sensillum lymph through cuticular pores, and function as chaperones that transport pheromones to the sex pheromone receptors localized on the dendritic membranes of ORNs. PBPs have six highly conserved cysteine residues that help stabilize the globular protein and the internal hydrophobic binding pocket [7].

Nerve impulses are generated by ORNs following the delivery of sex pheromones by the PBPs are mediated by transmembrane odorant receptors (ORs) located on the surface of neuron dendrite. Insect ORs dimerize with a highly conserved co-receptor termed the odorant receptor co-receptor (*Orco*) to form a ligand-gated ion channel [8]. Pheromones are released from carrier PBPs when lower pH microenvironments are encountered at the neuron surface, where selectivity of PBPs may be enhanced [9]. Pheromone ligand release by PBPs and subsequent binding to the ORco + OR_x_ complex will cause an electrical impulse to be generated [7]. Changes in the structure or function of these sexual communication pathway components form a hypothetical basis for the evolution of novel mechanisms of male attraction, and may be involved in establishment of sexual isolation between recently diverged species.

Species from the genus *Ostrinia* are models for the study of male sex pheromone detection systems. Different sex pheromone components are produced in the pheromone gland of females in the genus *Ostrinia* [10–13], but different ratios of *E*-11- and *Z*-11-tetradecenyl acetate (*E*11- and *Z*11-14:OAc) predominate among most species. One exception is found with females of the Asian corn borer (ACB), *O. furnacalis* (Guenée), that produce and emit pheromone components that are synthesized with the double bond at the twelfth carbonyl carbon [14]; *E*-12- and Z-12-tetradecenyl acetate (*E*12- and *Z*12-14:OAc). Mating between *O. furnacalis* and the closely related European corn borer (ECB), *O*. *nubilalis* (Hübner), occurs in the laboratory, but behavioral isolation and differences in response to sex pheromone may likely prevent high degrees of hybridization under natural conditions. The gene coding sequence of five PBPs were sequenced for both *O*. *furnacalis* and *O*. *nubilalis*, and with the exception of PBP3, the amino acid sequences between orthologs are highly conserved [15]. A significant overrepresentation of non-synonymous mutations predicted between PBP3 orthologs suggested a potential role in pheromone binding specificity between *E*11- and *Z*11-14:OAc, and *E*12- and *Z*12-14:OAc [15]. In this study, we expressed and compared binding affinities of the five *O*. *furnacalis* PBP paralogs for synthetic female *O*. *furnacalis* and *O*. *nubilalis* pheromones, and mutagenized four amino acid residues predicted to coordinate *E*12- and *Z*12-14:OAc pheromones within the hydrophobic binding pocket. The data presented in this study are important for understanding the functional residues that are involved in effecter binding by *Ostrinia* PBP3, but does not attempt to address the evolutionary changes that impart divergent function between *O. furacalis* compared to *O. nubilalis* PBPs. Regardless, the data presented here are important for understanding the residues involved in general sex pheromone binding, but additional research is required to investigate any residues involved in the selectivity of *Ofur*PBP3 compared to *On*PBP3.

## Materials

### RNA extraction and cDNA synthesis

A laboratory colony of *O. furnacalis* was maintained at the Institute of Plant Protection, Chinese Academy of Agricultural Sciences. Pupae were allowed to emerge in a netted cage at room temperature, and moths were fed a 10% honey solution. Antennae were dissected from three day old male moths, immediately frozen in liquid nitrogen and stored at −80 °C prior to use. Total RNA was extracted from *O. furnacalis* antennae using Trizol™ reagent (Invitrogen, USA) according to manufacturer instructions. RNA was then reverse transcribed into 1st strand cDNAs in a reaction primed with an oligodT_18_ oligonucleotide, and synthesized with Avian Myeloblastosis Virus (AMV) reverse transcriptase (Promega, USA) at 40 °C for 1 h. The reaction was stopped by incubation at 95 °C for 5 min and then cooling on ice for 20 min.

The cDNA sequences for five Asian corn borer PBP genes were previously reported by Allen and Wanner [15, NCBI accession number range GU828024 to GU828028], and were used to design primer pairs that amplified the in frame mature gene coding sequence for each gene (omitting the signal peptide). Each primer incorporated a novel 5-prime overhang with palindromic sites for cleavage by *Bam*HI (forward primer) or *Hind*III (reverse primer), which allowed for subsequent directional cloning into the restriction endonuclease sites of the multicloning site in the pET30a(+) expression vector (Novagen, Darmstadt, Germany). Briefly, PCR primers for the amplification of mature *Ofur*PBP1-*Ofur*PBP5 coding sequences were designed using Primer3 [16] with a minimum length of 19 nucleotides and a predicted melting temperature (Tm) of ≥58 °C. The 5-prime extensions that encompassed the *Bam*HI preceding the N-terminal CDS and *Hind*III site following the C-terminal coding region were inserted manually, the details of the primers was shown in Additional file 1: S1. The subsequent PCR reactions used 5 pmol of each primer used 1.0 μl of cDNA template diluted 1:10 with the high fidelity polymerase (Takara, Japan). Thermal cycling was performed on a Biometra T-Gradient thermocycler (Biometra, Göttingen, Germany) under the following conditions: 94 °C for 2 min 30 s, 35 cycles of 94 °C for 30 s, 58 °C for 30 s, and 72 °C for 1 min, and 72 °C for 10 min. An aliquot of each PCR product was separated on a 1.5% agarose gel containing 0.5 μg ml^−1^ ethidium bromides and visualized under UV illumination. PCR products were cloned into the pGEM-T vector (Promega, Madison, WI), and single clones were obtained after selection on LB agar plates containing 50 μg ml^−1^ ampicillin. For each *Ofur*PBP clone, plasmid DNA isolated and sequenced from the T7 primer binding site at Beijing SinoGenoMax Co., Ltd (Beijing, China) on an ABI 3730XL DNA Analyzer (Applied Biosystems, Foster City, CA, USA). Electropherogram data were compared to cDNA sequences of respective PBPs in GenBank accessions GU828024 to GU828028 previously published by [15] using Vector NTI software (Life Technologies, Grand Island, NY). Independent multiple sequence alignments were made using *Ofur*PBP1 to *Ofur*PBP5 sequences generated in this study, *O*. *furnacalis* (GU828024.1 to GU828028.1), *O*. *nubilalis E*-strain (GU828019.1 to GU828023.1), and *O*. *nubilalis Z*-strain (GU826166.1 to GU826170.1) using the Clustal W algorithm (gap opening penalty 15, gap extension penalty 6.66, weight matrix IUB, and transition weight of 0.5).

### Prokaryotic expression and purification

The pGEM plasmid inserts were excised using *Bam*HI and *Hind*III double digestion, excised from a 1% agarose gel and fragments purified using Nucleic Acid Purification Kit (Axygen, USA). The *Bam*HI and *Hind*III digestion products for *Ofur*PBP1 to *Ofur*PBP5 were then ligated individually into *Bam*HI and *Hind*III cut and dephosphorylated pET30a(+) vector by incubation with T4 Ligase (Promega) for 4 h at 16 °C. Recombinant pET30a(+) vectors containing inserts derived from the five pheromone binding proteins (*Ofur*PBP1-*Ofur*PBP5) were used to transform BL21(DE3) *Escherichia coli* cells by electroporation, and cells allowed to recover in SOC medium for 1 h at 37 °C. Transformed cells were selected by spread plating on LB agar plates containing 25 μg ml^−1^ kanamycin sulfates as described by the manufacturer. Positive clones were cultured, and plasmid DNA isolated and inserts sequenced using the T7 primer as described above.

Positive pET30a(+) clones containing correct insert DNA from each of the PBP sequences were incubated separately in fresh liquid LB medium containing 25 μg ml^−1^ kanamycin sulfate. Cultures were shaken at 37 °C until the optical density (OD) of each culture reached to 0.6–0.8, where OD was measured on a Shimadzu spectrophotometer (Shimadzu, Japan). Protein expression was induced in each culture by the addition of isopropyl-β-d-1-thiogalactopyranoside (IPTG) to a final concentration of 0.1 mM, followed by incubation on a 150 rpm shaker at 37 °C for 6 h. The cells were harvested by centrifugation at 7500×*g* for 15 min at 4 °C, chilled on ice, and treated with ultrasonic disruptor. After centrifugation, all five proteins were highly present in inclusion bodies. The protein pellets were suspended using previously published methods [17]. Proteins were purified by application of the lysate to Ni–NTA columns and purified as specified by the manufacturer (Qiagen, Germany), followed by digestion of eluted proteins with enterokinase to remove the His tag. Protein preparations were desalted by dialysis, quantified by means of the Bradford method [18], and purity verified by SDS-PAGE.

### Pheromone-binding affinity

Initial measures of the binding affinity of the fluorescent ligand *N*-phenyl-1-naphthylamine (1-NPN) to recombinant *Ofur*PBP1, *Ofur*PBP2, *Ofur*PBP3, *Ofur*PBP4 and *Ofur*PBP5 was determined by titrating to final concentrations of 1.0–12.0 µM 1-NPN with 2 µM of each protein in 1.0 ml 50 mM Tris–HCl, pH 7.4 and pH 4.0. The 1-NPN probe was excited at 337 nm, and emission spectra were recorded between 380 and 450 nm on a Shimadzu fluorescence spectrophotometer (Shimadzu, Japan). Each trial was run in triplicate, and Scatchard plots of the ratio of bound to unbound ligand were created using Microsoft Excel 2010. Competitive binding assays were performed with each of the *Ofur*PBPs using 1-NPN as the probe. Assay reactions were comprised of 2 µM of each *Ofur*PBP diluted individually in 2.0 ml 20 mM Tris–HCl, pH 7.4, which were then titrated with 1.0 mM increments sex pheromone diluted in methanol to a final concentrations of 1–6 µM. The synthetic standards that correspond to the sex pheromones of *O. furnacalis*, *O. nubilalis* and other lepidopteran species were purchased from Sigma-Aldrich (Shanghai, China; Additional file 2: S2). Excitation and emission spectra were recorded from triplicate assays as described above. The binding affinity of each purified *Ofur*PBP to the different sex pheromones was measured by calculation of binding constants via competitive fluorescence-binding assay. In brief, relative fluorescent intensities were analyzed by GraphPad Prism 5 software (GraphPad Software Inc, La Jolla, CA) and calculation was done using the equation: Ki = [IC50]/(1 + [1-NPN]/K_1-NPN_), where [1-NPN] is the free concentration of 1-NPN and K_1-NPN_ is the dissociation constant of the complex protein/1-NPN [19, 20]. Sex pheromone binding affinity with different PBPs was analyzed using One-way ANOVA in SAS 8.0 (SAS Institute Inc., Cary, NC, USA).

### Molecular modeling and ligand docking

The amino acids of *Ofur*PBP3 were submitted to SWISS-MODEL tool to automate homology-modeling [21]. The sequence of *Ofur*PBP3 was compared to all the known protein sequences and that had a high similarity with the target sequence on RCSB Protein Data Bank (PDB) (www.rcsb.org). The *Ofur*PBP3 used *Atra*PBP1 for a homology model [22]. The main pheromone components of *O. furnacalis*, *Z*12- and *E*12-14:OAc were used respectively in molecular docking with the simulated model of *Ofur*PBP3 based on the advanced docking program CDOCKER [23]. Molecular dynamics scheme based on CHARMm was used to dock ligands into a binding site in the CDOCKER program. Total interaction (*E*_total_), Van der Waals (*E*_vdw_), and electrostatic interaction energies (*E*_ele_) were calculated for all *Ofur*PBP3 residues predicted to be involved in the binding of intraspecific sex pheromone components.

### Site-directed mutagenesis

Four amino acids of *Ofur*PBP3 with some of the most negative estimated *E*_total_ values were mutated using Quick-change lightning site-directed mutagenesis kit (Stratagene, La Jolla, CA, USA); Ile to Asn at position 113 (I113A), m2 Phe to Ser at position 12 (F12S), m3 Ile to Asn at position 52 (I52N), and m4 Leu to Arg at position 94 (I94R). The sequence of each primers used to alter the aforementioned sites by nucleotide mismatch are shown in Additional file 3: S3. After initial denaturation at 94 °C for 5 min, the subsequent PCR followed with 30 cycles of 94 °C for 30 s, 56 °C for 30 s, and final extension at 72 °C for 1 min. Changes within site-directed mutant fragments were verified by cloning into the pGEM-T Easy vector (Promega) by Sanger sequencing. All mutated OfurPBPs proteins were expressed and used within in vitro binding assays as described above.

## Results

### RNA extraction and cDNA synthesis

Five amplified fragments predicted to contain the mature *Ofur*PBP1 to *Ofur*PBP5 gene coding sequences were successfully amplified by RT-PCR (approximate 450 bp in size; gel data not shown), cloned into the pGEM vector, and Sanger sequenced. Multiple nucleotide sequence alignments of these cloned *Ofur*PBP sequences showed 100% similarity with homologous regions of GenBank accessions for *O*. *furnacalis* PBP1 to PBP5 previously identified by Allen and Wanner [15]. One exception was predicted in a single nucleotide polymorphism at position 474 of *Ofur*PBP2 which changed a guanine (G) in GU828025.1 to an adenosine (A) in our cloned *Ofur*PBP2 sequence. This SNP on *OfurPBP*2 produced a synonymous mutation, such that the amino acid sequence was unchanged and the same as that reported by Allen and Wanner [15].

### Prokaryotic expression and purification

**Fig. 1:**
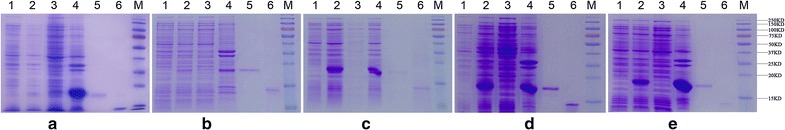
SDS-PAGE analysis of recombinant *Ostrinia furnacalis* pheromone binding proteins (PBPs) induced from *E. coli* clones carrying in frame insertions of OfurPBP1, OfurPBP2, OfurPBP3, OfurPBP4, or OfurPBP5 coding sequences in the pET30a(+) expression vector. Replicate gel lanes correspond to (*1*) cell culture prior to addition of isopropyl-β-d-1-thiogalactopyranoside (IPTG), (*2*) IPTG induced cells at O.D. 0.6–0.8, (*3*) supernatant after ultrasonic treatment and centrifugation, (*4*) pellet after ultrasonic treatment and centrifugation, (*5*) Ni–NTA column purified proteins, and (*6*) enterokinase digested products with His-tags cleaved. *M* corresponds to molecular weight marker. **a** OfurPBP1; **b** OfurPBP2; **c** OfurPBP3; **d** OfurPBP4; **e** OfurPBP5

Five mature *Ofur*PBP gene coding sequences were successfully excised from pGEM, and re-inserted into to the pET30a(+) expression vector. End sequencing of inserts from the T7 primer binding site flanking the pET30a(+) vector multicloning site confirmed the correct orientation and sequence of each *Ofur*PBP insert (data not shown). Each of the IPTG induced cultures derived from *E. coli* pET30a(+)-*OfurPBP* transformants showed high expression of a His-tagged fusion protein in the approximately 16–19 kDa size range (Fig. 1, lane 4), and subsequent purification of each on Ni–NTA columns successfully removed visible endogenous *E. coli* proteins (Fig. 1, lane 5). Digestion of purified *Ofur*PBPs fusion proteins with enterokinase (to remove the His-tags) resulted in decreased estimated molecular weights, which were less than the 15–17 kDa predicted for full-length lepidopteran PBPs containing leader peptides (Fig. 1, lane 6). This was expected since the cloned fragments were devoid of leader peptides and represent fully functional processed peptides. After analysis, most of the proteins were expressed in inclusion bodies. Bradford assays estimated the concentration of each purified *Ofur*PBP protein at ~1 to 2 μg ml^−1^, and extracts were used for the further experiments in this study.

### Pheromone-binding affinity

**Fig. 2:**
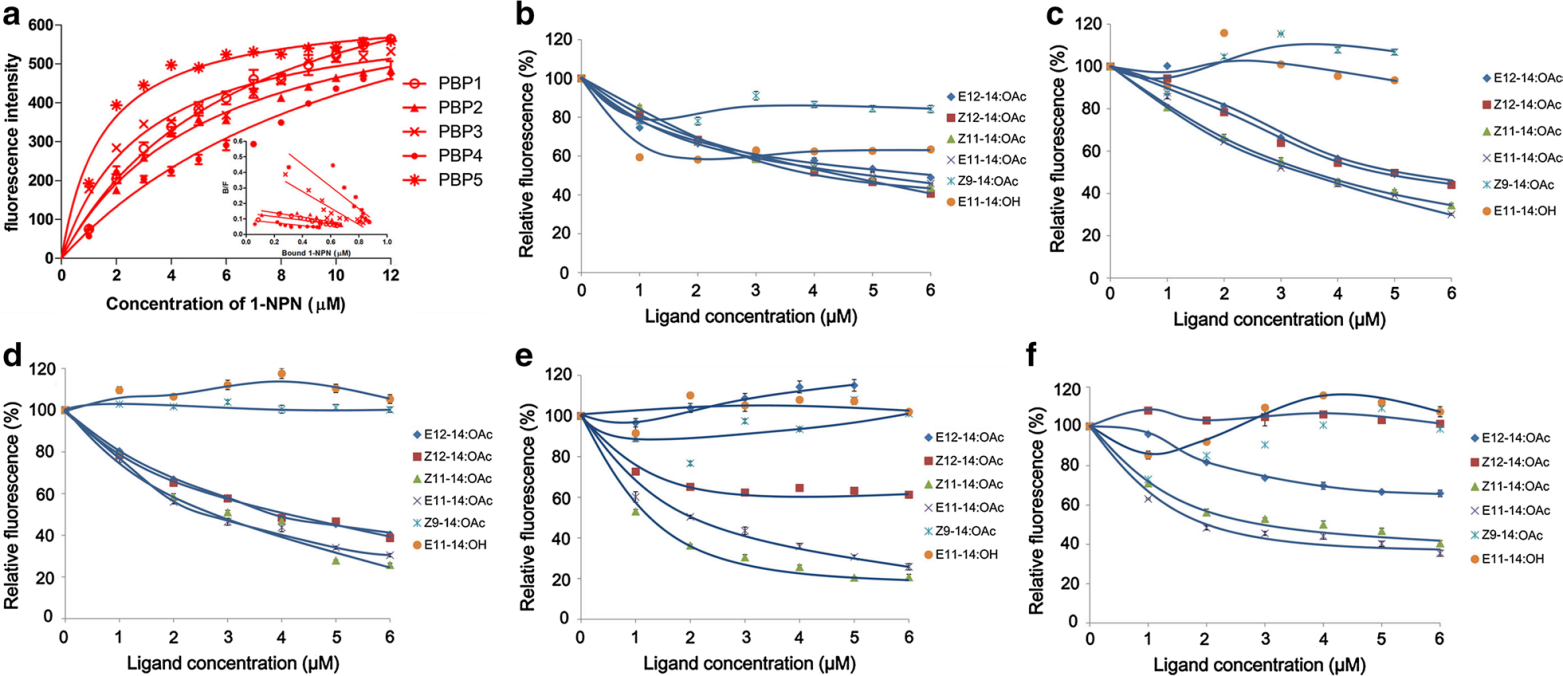
Binding curves. **a** Binding curves and Scatchard plots (*inset*) for fluorescent probe 1-NPN binding with purified *Ostrinia furnacalis* pheromone binding proteins (OfurPBPs) at pH 7.4. Competitive binding curves for different synthetic sex pheromone hydrocarbons (ligands) at pH 7.4 to each of the OfurPBPs, **b** OfurPBP1, **c** OfurPBP2, **d** OfurPBP3, **e** OfurPBP4, and **f** OfurPBP5. The different sex pheromones tested are listed in Additional file 2: S2

The general affinity of the 1-NPN reporter for each *Ofur*PBP was measured (Fig. 2a), and a regression of these titrated values were plotted within Scatchard plots and showed a linear relationship. Next, the binding specificity of 6 different *Ostrinia* pheromone components were tested against each recombinant *Ofur*PBP in competitive binding assays with 1-NPN as the reporter (Additional file 2: S2). The female sex pheromones, Z12- and *E*12-14:OAc are produced by *O. furnacalis* and *Z*11- and *E*11-14:OAc by *O*. *nubilalis* (see “Background” section). Competitive binding assay results showed that *Z*12- and *E*12-14:OAc bound most strongly with *Ofur*PBP3 (K_i_ ≤ 2.86 ± 0.04), and also showed affinity for *Ofur*PBP1 and *Ofur*PBP2 (K_i_ = 5.01 ± 0.14; Table 1; Fig. 2). Results also showed that *Z*12-14:OAc bound *Ofur*PBP5 weakly and E12-14:OAc had no detectable binding to *OfurPBP*4. In contrast, both *Z*11- and *E*11-14:OAc pheromones from *O*. *nubilalis* showed relatively high affinities for all five *OfurPBP*s (Table 1; Fig. 2), with estimates of Z11-14:OAc binding to *OfurPBP*4 being the highest for all those that were tested (K_i_ = 1.09 ± 0.05). Additionally, the *O. nubilalis* pheromone *E*11-14:OAc bound strongly to *Ofur*PBP5 (K_i_ = 1.14 ± 0.04), and showed a significantly greater affinity compared to either *Z12*- and *E*12-14:OAc from *O*. *furnacalis* (K_i_ = 6.80 ± 0.47). All five recombinant *Ofur*PBPs showed weak or lack of detectable binding to the interspecific lepidopteran sex pheromones, *Z*9-14:OH and *E*11-14:OH. However, under the pH 4.0, none of the *Ofur*PBPs showed any binding to the sex pheromones that were tested (data not shown).

**Table 1:** Competitive ligand binding assay results among five recombinant native *Ostrinia furnacalis* PBP orthologs (OfurPBP1 to 5) tested against six female sex pheromones found among species in the genus *Ostrinia*

	*Ostrinia nubilalis* female sex pheromones				*Ostrinia furnacalis* female sex pheromones				Other lepidopteran female sex pheromones			
	*Z*11-14:OAc		*E*11-14:OAc		*Z*12-14:OAc		*E*12-14:OAc		*Z*9-14:OH		*E*11-14:OH	
	IC_50_	K_i_	IC_50_	K_i_	IC_50_	K_i_	IC_50_	K_i_	IC_50_	K_i_	IC_50_	K_i_
OfurPBP1	4.69 ± 0.14	4.08 ± 0.12a	5.30 ± 0.02	4.61 ± 0.02a	4.88 ± 0.94	4.24 ± 0.82a	5.77 ± 0.16	5.01 ± 0.14a	–	–	–	–
OfurPBP2	3.49 ± 0.06	2.95 ± 0.05b	3.28 ± 0.01	2.77 ± 0.01b	4.96 ± 0.23	4.19 ± 0.09b	4.88 ± 0.10	4.12 ± 0.08b	–	–	16.43 ± 0.18	13.88 ± 0.15
OfurPBP3	3.27 ± 0.24	2.44 ± 0.18c	2.64 ± 0.15	1.97 ± 0.11c	3.83 ± 0.06	2.86 ± 0.04c	3.81 ± 0.02	2.85 ± 0.01c	–	–	–	–
OfurPBP4	1.18 ± 0.05	1.09 ± 0.05d	2.31 ± 0.57	2.13 ± 0.53c	9.97 ± 0.20	9.19 ± 0.18c	–	–	–	–	–	–
OfurPBP5	3.64 ± 0.28	2.18 ± 0.17e	1.90 ± 0.07	1.14 ± 0.04d	11.06 ± 1.13	6.64 ± 0.66d	11.34 ± 0.81	6.80 ± 0.47d	–	–	–	–

All assays performed at pH = 7.4. Values reported as μM concentrations of each pheromone component

IC_50_ is the ligand concentration displacing 50% of the fluorescent reporter; K_i_ is the binding constant calculated from the equation Ki = [IC_50_]/(1 + [1-NPN]/K_1-NPN_)

Mean ± SE, data in the same column followed by the same letters were not significantly different (P ≥ 0.05) according to LSD test. Dashes indicate undetectable interations

## 3D modeling and docking

**Fig. 3:**
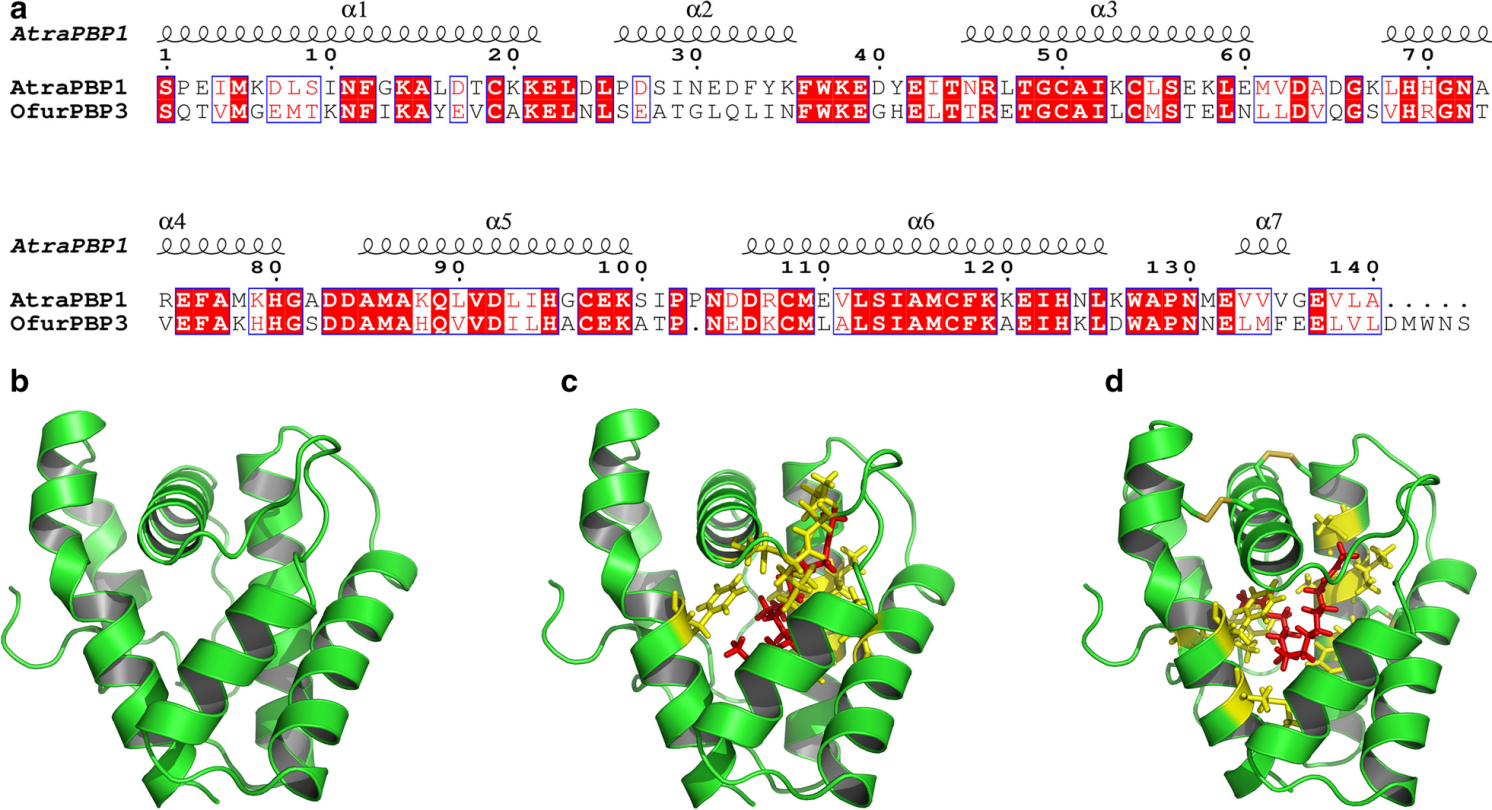
The predicted 3D structure of OfurPBP3 of *O. furnacalis* and docking with sex pheromones. **a** Sequence alignment between OfurPBP3 and AtraPBP1, and the predicted α-helical secondary structure elements are shown along the *top*. Strictly identical residues are highlighted in *white letters* with a *red background*, and are framed in *blue* residues are chemically identical or similar. Residues with similar physico-chemical properties are shown in *red letters*. The key amino acids used to mutated were marked by* black triangle*. **b** The predicted 3D structure of OfurPBP3. In silico predicted molecular docking between *Z*12-14:OAc (**c**) and *E*12-14:OAc (**d**) with OfurPBP3 are shown with respective pheromone components in *red* interacting amino acids are in *yellow*

The *Ofur*PBP3 was successfully modeling with *Atra*PBP1 by using of SWISS-MODEL tool (Fig. 3a). This figure visual displayed the 3-dimensional structure of *Ofur*PBP3. The QMEAN and GMQE were used to quality estimation, all the score reflected the expected accuracy of *Ofur*PBP3 built with the alignment and template of *Atra*PBP1. Based on the consideration of hydrophobicity at their binding site, ligand poses and consensus score programs were executed to evaluate binding pose affinities for the residues. Subsequently, optimal 3D binding conformations to the main sex pheromone components, *Z*12- and *E*12-14:OAc, were shown in Fig. 3c, d. The interaction energies *E*_total_, *E*_vdw_, and *E*_ele_ between *Ofur*PBP3 amino acids that interact with *Z*12- or *E*12-14:OAc were estimated (Table 2). Results indicated that Phe12 and Leu94 interacted most strongly with *Z*12-14:OAc. and Phe12 and Ile52 with *E*12-14:OAc.

**Table 2:** Interaction energies between *Ofur*PBP3 amino acid residues predicted to interact with *Z*12- and *E*12-14:OAc

Residues	*Z*12-14: OAc			*E*12-14: OAc		
	*E*_total_	*E*_vdw_	*E*_*eie*_	*E*_total_	*E*_vdw_	*E*_*eie*_
Met5	−0.640547	−0.59189	−0.04866	−0.25078	−0.48178	0.231002
Met8	−2.03208	−1.93288	−0.0992	−2.08551	−1.95942	−0.12609
Thr9	−0.725306	−0.69731	−0.028	−0.99693	−0.94188	−0.05505
Phe12^a^	−3.44675	−3.59075	0.144002	−6.12711	−6.37345	0.246336
Trp37	−0.265347	−0.34278	0.077433	−3.00745	−2.93172	−0.07573
Ile52^a^	−2.06826	−1.91544	−0.15282	−1.04584	−1.05974	0.013902
Leu53	−0.602925	−0.69798	0.095052	−3.66956	−3.21571	−0.45385
Ser56	−2.3887	−2.1026	−0.2861	−1.34568	−1.50106	0.155383
Leu61	−2.80413	−3.02047	0.216336	−1.89133	−2.01921	0.127883
Thr73	−0.502607	−0.6027	0.100092	−0.85839	−0.52394	−0.33445
Phe76	−1.22346	−1.2791	0.055643	−1.40333	−1.4029	−0.00043
Val90	−0.59213	−1.00189	0.40976	−0.71638	−0.44384	−0.27254
Leu94^a^	−3.31952	−3.46491	0.145392	−2.20259	−2.17874	−0.02385
Ala110	−2.27602	−2.23154	−0.04448	−2.29651	−1.83678	−0.45973
Ile113^a^	−4.09863	−4.16508	0.066446	−2.37414	−2.46977	0.095626
Ala114	−2.21266	−2.52149	0.308827	−2.80034	−2.36719	−0.43315
Phe117	−2.81675	−3.782	0.965255	−3.08384	−3.35722	0.273382
Met133	−0.604454	−0.6274	0.022947	−2.5398	−2.6934	0.153598

*E*_total_: total interaction energy; *E*_vdw_: Van der Waals energy; *E*_ele_: electrostatic interaction energy

^a^Residues chosen for site-directed mutagenesis

### Site-directed mutagenesis

Based on the molecular modeling and docking results four amino acid residues with among the highest *E*_total_ were chosen (Phe 12, Ile52, Leu94 and Ile113), and in vitro mutagenesis altered *Ofur*PBP3 mutants F12S, I52N, I94R, and I113N verified from Sanger sequencing results (Additional file 4: S4). Subsequent competitive binding assays demonstrated that all *Ofur*PBP3 mutants showed reduced affinities towards *Z*11- and *E*11-14:OAc (8.53–20.11 μM) and *Z*12- and *E*12-14:OAc (4.89–18.77 μM; Table 3) compared to the wild-type *Ofur*PBP3. Among the mutants, I113N and F12S had the least dramatic effect on affinity towards *Z*12-14:OAc (~2-fold reduction). Surprisingly I113N showed a nearly 8.25-fold reduction in *Z*11-14:OAc binding compared to the wildtype, and represented the greatest degree of change among all the assays. All mutated *Ofur*PBPs showed the capacity to bind the sex pheromones components of other lepidopteran females, *Z*9-14:OH and *E*11-14:OH (Table 3), compared to the wildtype which failed to demonstrate this function. In particular, I113N demonstrated the ability to bind *Z*9:14-OH, which is a known antagonist of male response to *E*11- and *Z*11-14:OAc as well as *E*12- and *Z*12-14:OAc (see “Discussion” section).

**Table 3:** Competitive ligand binding assay results from four *Ostrinia furnacalis* PBP3 (OfurPBP3) mutants generated by site-directed mutagenesis of Ile113 to Asn113 (M1: I113N), Phe12 to Ser12 (M2: F12S), Ile52 to Asn52 (M3: I52N), and Ile94 to Arg94 (M4: I94R)

	*Ostrinia nubilalis* female sex pheromones				*Ostrinia furnacalis* female sex pheromones				Other lepidopteran female sex pheromones			
	*Z*11-14:OAc		*E*11-14:OAc		*Z*12-14:OAc		*E*12-14:OAc		*Z*9-14:OH		*E*11-14:OH	
	IC_50_	Ki	IC_50_	Ki	IC_50_	Ki	IC_50_	Ki	IC_50_	Ki	IC_50_	Ki
*Ofur*PBP3	3.27 ± 0.24	2.44 ± 0.10c	2.64 ± 0.15	1.97 ± 0.07b	3.83 ± 0.06	2.85 ± 0.02b	3.81 ± 0.02	2.84 ± 0.01b	–	–	–	–
M1 I113 N	27.81 ± 9.01	20.11 ± 3.76a	11.00 ± 3.86	5.57 ± 1.61ab	6.78 ± 2.27	4.91 ± 0.95ab	8.03 ± 1.77	11.60 ± 3.81ab	6.14 ± 1.18	4.42 ± 0.85	10.38 ± 1.20	8.08 ± 0.33
M2 F12S	10.58 ± 1.24	6.53 ± 0.57bc	8.17 ± 0.28	6.53 ± 0.13ab	6.09 ± 0.57	4.89 ± 0.27ab	7.47 ± 0.87	6.02 ± 0.40ab	9.52 ± 1.28	7.69 ± 1.02	12.35 ± 2.58	9.87 ± 2.06
M3 I52 N	13.29 ± 2.16	12.96 ± 1.04ab	14.97 ± 0.10	12.48 ± 0.05a	11.92 ± 4.37	10.65 ± 2.10a	18.77 ± 3.30	13.03 ± 1.59ab	18.33 ± 1.31	16.35 ± 1.09	15.45 ± 2.50	12.87 ± 2.09
M4 I94R	9.83 ± 0.76	8.71 ± 0.39bc	12.65 ± 2.11	13.78 ± 2.13a	10.59 ± 1.71	9.50 ± 0.88ab	17.40 ± 3.49	15.42 ± 1.79a	12.01 ± 2.24	10.81 ± 1.99	11.09 ± 2.50	10.47 ± 2.22

Mutant and wildtype OfurPBP proteins were assayed against six female sex pheromones found among species in the genus *Ostrinia*. All assays performed at pH = 7.4. Values reported as μM concentrations of each pheromone component

IC_50_ is the ligand concentration displacing 50% of the fluorescent reporter; K_i_ is the binding constant calculated from the equation K_i_ = [IC_50_]/(1 + [1-NPN]/K_1-NPN_)

Mean ± SE, date in the same column followed by the same letters were not significantly different (P ≥ 0.05) according to LSD test. Dashes indicate undetectable interations

## Discussion

We showed that the five recombinant *Ofur*PBPs have a range of binding affinities with synthetic *O*. *furnacalis* sex pheromones, and that *Ofur*PBP2 and *Ofur*PBP3 bind Z12- and *E*12-14:OAc with the greatest affinity (Table 1; Fig. 2). These binding results might suggest that female *O*. *furnacalis* pheromones are bound strongly to male-antennal expressed *Ofur*PBP2 and *Ofur*PBP3 in vivo, and that these two chaperones could play a role in the specific transport of *Z*12- and *E*12-14:OAc within the sensillar lymph [15]. The highly biased expression of *Ofur*PBP2 and *Ofur*PBP3 transcripts in the *Ostrinia* male compared to female antennae has been used to suggest possible roles in the binding of female sex pheromones [15], and the latter hypothesis was corroborated for the first time by our data (Table 1). The correlation between PBP expression level and molecular function was further corroborated by *Ofur*PBP4 and *Ofur*PBP5, wherein both show female biased expression (e.g. low expression in male antennae; Allen and Wanner [15]) and have weak or undetectable binding affinities towards *E*12- and *Z*12-14:OAc (Table 1). A difference in pheromone binding affinity has been previously shown for PBP1 from *A*. *polyphemus* and *A*. *pernyi* which were both bound strongly by *E*4,9*Z*-14:Ac, but PBP2 and PBP3 from these species preferentially bound *E*6,11Z-16:Ald and *E*4,9*Z*-14:Ac, respectively [24]. Analogously, PBP1 and PBP2 from *Lymantria dispar* male antennae respectively bind specifically to (+) and (−) enantiomers of the pheromone released by conspecific females [25], which suggest that PBP gene family members can develop variant molecular functions and that our observed differences between *Ofur*PBP2 and *Ofur*PBP3 compared to *Ofur*PBP4 and *Ofur*PBP5 may not be surprising. Furthermore, the binding affinities of *Ofur*PBP1, *Ofur*PBP2, and *Ofur*PBP3 to cognate pheromone components estimated in this study are comparable to analogous Ki values estimated for *Helicoverpa armigera* PBPs (Ki values 1.2–5.2 μM) and *H*. *assulta* (0.7–4.1 μM) to their corresponding sex pheromone [26]. Similarly, the binding affinities of the cockroach *Leucophaea maderae* PBP, PBPLma, to their sex pheromones 3-hydroxy-butan-2-on or butane-2,3-diol were 3.8 and 2.5 μM, respectively [27].

In contrast to predictions made by Allan and Wanner (2010), *Ofur*PBP3 did not show specific affinity towards *Z*12- and *E*12-14:OAc, but instead demonstrated a nearly equal affinity for *Z*11- and *E*11-14:OAc emitted by *O. nubilalis* females. Moreover, all *Ofur*PBPs showed a comparatively strong affinity towards *Z*11- and *E*11-14:OAc, including observation of strong binding by *Ofur*PBP4 and *Ofur*PBP5. These ligand affinities are in direct opposition to species responses [28], which might suggest that *Ofur*PBPs bind to *Z*11- and *E*11-14:OAc does not preclude a role in male sexual response, but only in potential for binding these components within the sensillar lymph. Transcripts encoding PBPs have previously been found in non-pheromone sensitive female antennae and male sensilla [29, 30], which suggests that PBPs may have evolved alternate roles such as host plant recognition [31]. This notion was reinforced by evidence that compounds structurally similar to cognate pheromones can be bound by *Bombyx mori* PBP1 [32], and further by observations that of sex pheromones and plant volatiles can interact within the same peripheral sensory pathways [33, 34]. This altogether suggests that binding compounds or interspecific pheromone components to a PBP does not preclude subsequent interaction with the ORco + OR_x_ complex resulting in sexual response. Grosse-Wilde et al. [35] showed that pheromone agonists bound to different PBP paralogs and lead to different responses by male sexually-responding ORco − OR_x_ receptors, such that members of the PBP gene family may have evolved specific interactions at different ORco − OR_x_ complexes. The *O*. *furnacalis* OR3 (OfOR3) was shown to specifically respond to *Z*12-14:OAc and *E*12-14:OAc [36]. The selectivity of PBP for delivery of these pheromone components to OfOR3 was not investigated in this or any other previous studies, but may provide crucial information regarding the evolution of species-specific male sexual response. Regardless, our results are the first to demonstrate that male antennal expressed *Ofur*PBP2 and 3 are capable of binding the intraspecific female pheromone components *Z*12-14:OAc and *E*21-14:OAc.

A change in the 3-dimensional conformation of PBPs occurs following a shift from neutral to acidic pH environments, and is associated with binding and release of cognate pheromone ligands. Crystal structures of pheromone-bound PBPs indicated that the C-terminal amino acid sequence remains linear under neutral pH [37], whereas this C-terminal region forms an alpha-helix in acidic conditions [alpha helix 7, α7; 37, 38]. The more linear conformation with 6 alpha helices was used to predict the 3D structure of *Ofur*PBP3 (Fig. 3b), wherein 18 residues within the hydrophobic binding pocket were predicted to interact with *Z*12-14:OAc and *E*21-14:OAc (Table 2). This pH-dependent change is also accompanied by alteration of PBP 3-dimensional structure where salt bridges are formed between two Asp residues on the C-terminal α7 helix and two corresponding deprotonated His residues in the pheromone binding pocket at low pH. Thus, the pH-dependent alpha-helix interactions with the pheromone binding pocket competes with sex pheromone ligands, and is a key component for PBP pheromone carrier and delivery functions. Specifically, neutral pH in sensillar lymph may place PBPs into a competent pheromone-binding state, where these chaperones are capable of transporting hydrophobic pheromone hydrocarbons.

Residues of *Ofur*PBP3 potentially involved during the binding of intraspecific pheromones at neutral pH binding are important for understanding function within a species, and the *E*_Total_ derived from CDOCKER predicted the 18 key amino acids which may interact with *Z*12- and *E*12-14:OAc ligands (Table 2). Predictions from homology-based in silico structural modeling can be unreliable [21] and translation into biological systems can be bolstered by experimental validation. Thus, site-directed mutagenizes of four residues with the highest *E*_total_ values were made in four separate mutant *Ofur*PBPs. Comparisons between wildtype and mutant *Ofur*PBPs clearly demonstrated the negative affects on binding affinities following change of native residues to those with different polar properties and suggest that Phe12, Ile52, Ile94, and Ile113 are important for the efficient binding of cognate sex pheromones. Interestingly, after mutation, the *Ofur*PBPs showed weak binding affinity towards other lepidopteran female sex pheromone, Z9-14:OH and *E*11-14:OH, that was not observed with the wild type *Ofur*PBP3. These differences may occur either through direct interaction with the bound hydrocarbons, or by stabilization of the hydrophobic binding pocket. The affects of induced amino acid changes on the overall 3D structure and function of a protein remain largely unknown [39]. This suggests that even single amino acid changes might alter PBP conformation in unforeseen ways, such that further research, such as using x-ray or NMR to analyze the structure of native and mutant binding proteins are likely required. Although our current study demonstrated the ability of *Ofur*PBPs to bind inter and intraspecific sex pheromones, further research is likely required to fully understand the structural, chemical, and environmental factors that may influence any specific interactions between PBPs and their cognate sex pheromones.

## Conclusions

Our research provides crucial functional information regarding the specificity of PBPs in an *Ostrinia* moth species, which is a group that has emerged as a model for the study of sexual communication systems in Lepidoptera. This study provides the first biochemical evidence that *Ofur*PBP2 and *Ofur*PBP3 bind *Z*12- and *E*12-14:OAc with high affinity. Moreover, this current work demonstrates that despite prior evidence for directional selection and predicted divergent function between *On*PBP3 and *Ofur*PBP3, the latter shows a nearly equal affinity to *O. furnacalis* female *Z*12- and *E*12-14:OAc and *O. nubilalis Z*11- and *E*11-14:OAc. The four amino acids, Ser12, Asn52, Arg94, and Asn113 may play a role in binding of sex pheromone of PBP3, but cannot be interpreted in the context of species-specific binding since we only mutagenized sites predicted to be important in protein interactions with pheromone ligands. Our results suggest that overall individual *O. furnacalis* PBPs may be capable of equivalent binding of both intra- and inter-specific female sex pheromone components in vitro, and therefore could suggest that molecular discrimination in male response might reside within the behavioral pathway. Additional research is undoubtedly required to both dissect the molecular functions of PBPs in concert with cognate ORs with respect to ligand binding as well as the mechanisms that transmit neuronal signals which result in differential male responses to female emitted pheromones.

## Additional files

**Additional file 1: S1.** The primers used for the PBPs expression. The lists of primers used for PBPs expression, and the digestion sites were underlined.

**Additional file 2: S2.** The message of sex pheromones used in binding assay. The sex pheromones messages used in binding experiment, including the molecular weight, purity and company.

**Additional file 3: S3.** The primers used for site-directed mutants. The lists of primers used for site-directed mutants, and the digestion sites were underlined.

**Additional file 4: S4.** The sequences of the wild type OfurPBP3 and the mutants. The nucleotide sequences of wild type OfurPBP3 and the mutants (OfurPBP3-m1, OfurPBP3-m2, OfurPBP3-m3 and OfurPBP3-m4).
