# Supplementary material for: Binding affinity of five PBPs to Ostrinia sex pheromones
Source: BMC Mol Biol. 2017 Feb 7;18:4. doi: 10.1186/s12867-017-0079-y (PMC5296967; doi:10.1186/s12867-017-0079-y)
Supplement: Supplementary file 1 — Additional file 1: S1. The primers used for the PBPs expression. The lists of primers used for PBPs expression, and the digestion sites were underlined. [file 12867_2017_79_MOESM1_ESM.doc]

Supplementary material S1: the primers used for the PBPs expression

| **Primer names** | **Primer sequences** | **Restriction enzyme** |
| --- | --- | --- |
| OfurPBP1-F | CGGATCCTCGCAAGATGTGATGAAAC | BamHI |
| OfurPBP1-R | CGAAGCTTTTAAACTTCAGCCAACACCTCG | Hind III |
| OfurPBP2-F | CGGATCCTCACAAGCAGTGATGAAAG | BamHI |
| OfurPBP2-R | GCAAGCTTTCATTGCTTCATTTCGGCC | Hind III |
| OfurPBP3-F | CAGGATCCTCACAAACAGTGAT | BamHI |
| OfurPBP3-R | CGCAAGCTTTCATGAATTCCAC | Hind III |
| OfurPBP4-F | GCGGATCCTCTGAAGAACTTATGAC | BamHI |
| OfurPBP4-R | CAAGCTTTTAAGCCATCCCAGCCA | Hind III |
| OfurPBP5-F | CTGGATCCGAAATGGTTCCTGA | BamHI |
| OfurPBP5-R | CGAAGCTTTCAAACTTCTATAACTTCGG | Hind III |
