# Supplementary material for: Binding affinity of five PBPs to Ostrinia sex pheromones
Source: BMC Mol Biol. 2017 Feb 7;18:4. doi: 10.1186/s12867-017-0079-y (PMC5296967; doi:10.1186/s12867-017-0079-y)
Supplement: Supplementary file 2 — Additional file 2: S2. The message of sex pheromones used in binding assay. The sex pheromones messages used in binding experiment, including the molecular weight, purity and company. [file 12867_2017_79_MOESM2_ESM.doc]

Supplementary material S2: The message of sex pheromones used in binding assay

| Sex pheromones | Abbreviation | Chemical formula | Molecular weight | Purity | Company |
| --- | --- | --- | --- | --- | --- |
| (Z)-11-Tetradecenyl acetate | Z11-14: OAc | C_16_H_30_O_2_ | 254 | > 95% | Sigma-Aldrich |
| (E)-11-Tetradecenyl acetate | E11-14: OAc | C_16_H_30_O_2_ | 254 | > 95% | Sigma-Aldrich |
| (Z)-12-Tetradecenyl acetate | Z12-14: OAc | C_16_H_30_O_2_ | 254 | > 95% | Sigma-Aldrich |
| (E)-12-Tetradecenyl acetate | E12-14: OAc | C_16_H_30_O_2_ | 254 | > 95% | Sigma-Aldrich |
| (Z)-9-Tetradecenol | Z9-14: OH | C_14_H_28_O | 212 | > 98% | Sigma-Aldrich |
| (E)-11-Tetradecenol | E11-14:OH | C_14_H_28_O | 212 | > 98% | Sigma-Aldrich |
