# Supplementary material for: Binding affinity of five PBPs to Ostrinia sex pheromones
Source: BMC Mol Biol. 2017 Feb 7;18:4. doi: 10.1186/s12867-017-0079-y (PMC5296967; doi:10.1186/s12867-017-0079-y)
Supplement: Supplementary file 3 — Additional file 3: S3. The primers used for site-directed mutants. The lists of primers used for site-directed mutants, and the digestion sites were underlined. [file 12867_2017_79_MOESM3_ESM.doc]

Supplementary material S3: the primers used for site-directed mutants

| **Primer names** | **Primer sequences** | **Mutated amino acids** |
| --- | --- | --- |
| OfurPBP3-Fm1 | GACAAAGAATTCCATAAAAGCCTACG | Phe12 (TTC) to Ser (TCC) |
| OfurPBP3-Fm1 | CGTAGGCTTTTATGGAATTCTTTGTC |  |
| OfurPBP3-Fm2 | GTTGAGCAACGCCATGTGCTTCAA | Ile113 (ATC) to Asn (AAC) |
| OfurPBP3-Fm2 | TTGAAGCACATGGCGTTGCTCAAC |  |
| OfurPBP3-Fm3 | AGGATGCGCCAACCTCTGCATGT | Ile52 (ATC) to Asn (AAC) |
| OfurPBP3-Fm3 | ACATGCAGAGGTTGGCGCATCCT |  |
| OfurPBP3-Fm4 | GTAGACATTCGCCATGCTTGTG | Leu94 (CTC) to Arg (CGC) |
| OfurPBP3-Fm4 | CACAAGCATGGCGAATGTCTAC |  |
