# Supplementary material for: Binding affinity of five PBPs to Ostrinia sex pheromones
Source: BMC Mol Biol. 2017 Feb 7;18:4. doi: 10.1186/s12867-017-0079-y (PMC5296967; doi:10.1186/s12867-017-0079-y)
Supplement: Supplementary file 4 — Additional file 4: S4. The sequences of the wild type OfurPBP3 and the mutants. The nucleotide sequences of wild type OfurPBP3 and the mutants (OfurPBP3-m1, OfurPBP3-m2, OfurPBP3-m3 and OfurPBP3-m4). [file 12867_2017_79_MOESM4_ESM.doc]

OfurPBP3

TCACAAACAGTGATGGGAGAAATGACAAAGAATTTCATAAAAGCCTACGAAGTGTGTGCAAAAGAGCTCAACCTGTCCGAGGCCACAGGATTACAACTGATCAACTTTTGGAAGGAGGGCCACGAGTTGACGACTCGCGAGACAGGATGCGCCATCCTCTGCATGTCGACCGAGCTGAACCTGCTGGACGTTCAGGGGAGTGTGCACCGCGGGAACACTGTTGAGTTCGCCAAGCACCATGGCTCTGACGACGCAATGGCTCACCAAGTGGTAGACATTCTCCATGCTTGTGAAAAGGCTACACCCAACGAGGACAAGTGCATGTTGGCGTTGAGCATCGCCATGTGCTTCAAGGCCGAGATACACAAGCTGGACTGGGCACCCAACAACGAGCTGATGTTTGAGGAGTTGGTGTTAGATATGTGGAATTCATGA

OfurPBP3-m1

TCACAAACAGTGATGGGAGAAATGACAAAGAATTCCATAAAAGCCTACGAAGTGTGTGCAAAAGAGCTCAACCTGTCCGAGGCCACAGGATTACAACTGATCAACTTTTGGAAGGAGGGCCACGAGTTGACGACTCGCGAGACAGGATGCGCCATCCTCTGCATGTCGACCGAGCTGAACCTGCTGGACGTTCAGGGGAGTGTGCACCGCGGGAACACTGTTGAGTTCGCCAAGCACCATGGCTCTGACGACGCAATGGCTCACCAAGTGGTAGACATTCTCCATGCTTGTGAAAAGGCTACACCCAACGAGGACAAGTGCATGTTGGCGTTGAGCATCGCCATGTGCTTCAAGGCCGAGATACACAAGCTGGACTGGGCACCCAACAACGAGCTGATGTTTGAGGAGTTGGTGTTAGATATGTGGAATTCATGA

OfurPBP3-m2

TCACAAACAGTGATGGGAGAAATGACAAAGAATTTCATAAAAGCCTACGAAGTGTGTGCAAAAGAGCTCAACCTGTCCGAGGCCACAGGATTACAACTGATCAACTTTTGGAAGGAGGGCCACGAGTTGACGACTCGCGAGACAGGATGCGCCATCCTCTGCATGTCGACCGAGCTGAACCTGCTGGACGTTCAGGGGAGTGTGCACCGCGGGAACACTGTTGAGTTCGCCAAGCACCATGGCTCTGACGACGCAATGGCTCACCAAGTGGTAGACATTCTCCATGCTTGTGAAAAGGCTACACCCAACGAGGACAAGTGCATGTTGGCGTTGAGCAACGCCATGTGCTTCAAGGCCGAGATACACAAGCTGGACTGGGCACCCAACAACGAGCTGATGTTTGAGGAGTTGGTGTTAGATATGTGGAATTCATGA

OfurPBP3-m3

TCACAAACAGTGATGGGAGAAATGACAAAGAATTTCATAAAAGCCTACGAAGTGTGTGCAAAAGAGCTCAACCTGTCCGAGGCCACAGGATTACAACTGATCAACTTTTGGAAGGAGGGCCACGAGTTGACGACTCGCGAGACAGGATGCGCCAACCTCTGCATGTCGACCGAGCTGAACCTGCTGGACGTTCAGGGGAGTGTGCACCGCGGGAACACTGTTGAGTTCGCCAAGCACCATGGCTCTGACGACGCAATGGCTCACCAAGTGGTAGACATTCTCCATGCTTGTGAAAAGGCTACACCCAACGAGGACAAGTGCATGTTGGCGTTGAGCATCGCCATGTGCTTCAAGGCCGAGATACACAAGCTGGACTGGGCACCCAACAACGAGCTGATGTTTGAGGAGTTGGTGTTAGATATGTGGAATTCATGA

OfurPBP3-m4

TCACAAACAGTGATGGGAGAAATGACAAAGAATTTCATAAAAGCCTACGAAGTGTGTGCAAAAGAGCTCAACCTGTCCGAGGCCACAGGATTACAACTGATCAACTTTTGGAAGGAGGGCCACGAGTTGACGACTCGCGAGACAGGATGCGCCATCCTCTGCATGTCGACCGAGCTGAACCTGCTGGACGTTCAGGGGAGTGTGCACCGCGGGAACACTGTTGAGTTCGCCAAGCACCATGGCTCTGACGACGCAATGGCTCACCAAGTGGTAGACATTCGCCATGCTTGTGAAAAGGCTACACCCAACGAGGACAAGTGCATGTTGGCGTTGAGCATCGCCATGTGCTTCAAGGCCGAGATACACAAGCTGGACTGGGCACCCAACAACGAGCTGATGTTTGAGGAGTTGGTGTTAGATATGTGGAATTCATGA
